# Public Awareness and Knowledge of Sepsis: A Cross-Sectional Survey of Adults in the Western Region of Saudi Arabia

**DOI:** 10.7759/cureus.49102

**Published:** 2023-11-20

**Authors:** Yasser Alnofaiey, Shahad Mansour Alharthi, Raghad M Alhulayfi, Maram M Alqurashi, Renad M Alsufyani, Ghadi M Alamri

**Affiliations:** 1 Internal Medicine, College of Medicine, Taif University, Taif, SAU; 2 Medicine, College of Medicine, Taif University, Taif, SAU

**Keywords:** immunodeficiency, bacteria, shock, infection, sepsis

## Abstract

Background: Sepsis is a condition in which the body responds improperly to harmful microorganisms and infections, which triggers a chain reaction throughout the body. It is a life-threatening medical emergency. Infections that lead to sepsis, in most cases, start in the lungs, urinary tract, skin, or gastrointestinal tract. Without timely treatment, sepsis rapidly leads to multi-organ failure and death. The risk factors for sepsis include a weakened immune system, being 65 years of age or older, and chronic medical conditions including diabetes, lung disease, and kidney disease. A thorough understanding of the warning signs and symptoms, as well as prompt medical attention, are required for successful sepsis management. Early detection, antimicrobial therapy, and intensive supportive care are the cornerstones of the best sepsis management.

Aim: This study aimed to assess the public awareness and knowledge of sepsis in the Western region of Saudi Arabia.

Methods: This was a cross-sectional study conducted from the beginning of June till the end of August 2023, among 425 Saudi adults in the Western region above the age of 18 years. Data collection was carried out by administering a questionnaire through an online platform. The questionnaire included demographic information, risk factors, symptoms, protective factors, and the source of information about sepsis. The data collected were reviewed, coded, and then fed into IBM SPSS Statistics software for Windows, Version 21 (released 2012; IBM Corp., Armonk, NY).

Results: The study found that almost half (47.8%) of participants were aware of bacterial septicemia, with the most common sources of awareness being the internet (48.3%) and healthcare professionals (47.8%). Low immunity (75.9%) and infection (39.4%) have been the most frequently reported risk factors. Fever (31.5%) was the most commonly reported symptom. Participants from Yanbu and Jeddah and those in the healthcare sector had significantly higher awareness levels. The majority of participants (93.1%) had poor knowledge about the risk factors and symptoms of septicemia, with only 3.4% having fair or good knowledge. Sociodemographic characteristics did not significantly affect knowledge about septicemia (P>0.05).

Conclusion: In summary, this study reveals that while awareness of bacterial septicemia is relatively high among the sample population, knowledge about the risk factors and symptoms of the condition is low. The internet, healthcare professionals, and social media play significant roles in raising awareness about bacterial septicemia. Participants from Yanbu and Jeddah, students, and healthcare sector workers had higher awareness levels compared to other groups. However, overall knowledge levels regarding bacterial septicemia were poor across various demographic characteristics. This highlights the need for targeted educational interventions to improve knowledge and understanding of septicemia and its risk factors and symptoms among the general population. Further research is needed to identify the reasons for low knowledge levels and develop effective strategies to improve awareness about bacterial septicemia.

## Introduction

In 1992, sepsis was defined as an infection-induced systemic inflammatory response to infection [1]. Sepsis, according to the 2016 definition, is defined as "life-threatening organ dysfunction caused by an uncontrolled host response to infection" [1]. The new definition emphasizes homeostasis imbalance and infection response, as well as mortality and the importance of detecting sepsis as early as possible [2]. A thorough understanding of the warning signs and symptoms, as well as prompt medical attention, are required for successful sepsis management. Early detection, antimicrobial therapy, and intensive supportive care are the cornerstones of the best sepsis management and treatment.

In 1991, the first definition of sepsis was developed at a consensus conference in Chicago by members of the Society of Critical Care Medicine and the American College of Chest Physicians to help clinicians improve detection and allow for early therapeutic intervention [3, 4]. Following that, clinical terms such as "systemic inflammatory response syndrome", "severe sepsis", "sepsis", "septic shock", and "multiple organ dysfunction syndromes" were introduced [4].

Sepsis was defined as “two or more systemic inflammatory responses”, while severe sepsis was clinical sepsis accompanied by organ dysfunction, hypotension, or hypoperfusion. Septic shock is defined as a clinical scenario characterized by profound circulatory, cellular, and metabolic abnormalities associated with a high risk of mortality [5]. Several organizations have been formed to prevent sepsis and raise public awareness about it. The Global Sepsis Alliance is the leading organization in the United States, with a vision of a world without sepsis and a mission to save lives and reduce suffering by raising awareness of sepsis as an emergency [6].

Every year, nearly 50 million people worldwide are affected by sepsis [7]. The guideline published in 2016 defines sepsis as having a respiratory rate of at least 22 breaths per minute, altered mental status, and a systolic blood pressure of at least 100 mmHg [7]. Successful sepsis management requires a thorough understanding of the warning signs and symptoms of a medical emergency that requires prompt medical attention. Based on the 2016 guideline, the best sepsis management relies on early detection, antimicrobial therapy, and intensive supportive care [8]. Previous studies on sepsis detection have revealed gaps in sepsis recognition and management among medical and paramedical personnel [9]. The proportion of the general public who had heard of sepsis varied greatly. Across the globe, ranging from 4% in France [10] to 88% in Germany [11]. Most public-focused studies found that less than half of the participants were reportedly aware of sepsis, despite the fact that public awareness appeared to have gradually improved over time [9]. In Saudi Arabia, studies that assess the public's knowledge about sepsis are scarce.

## Materials and methods

Study design and participants

We utilized a cross-sectional study design. The study was conducted from the beginning of June till the end of August 2023 among adults aged 18 years and older in the Western region of Saudi Arabia to assess the level of awareness and knowledge of sepsis and to determine the factors that affect this knowledge.

Inclusion and exclusion criteria

The study included adult (above 18 years old) Saudi residents in the Western region of Saudi Arabia, both male and female, who presented signs and symptoms of sepsis and who consented to participate in the study. This study excluded residents from other regions of Saudi Arabia, patients without signs and symptoms of sepsis, and those below the age of 18 years.

Sample size

All participants who met the inclusion criteria and fully completed the questionnaire were considered study participants. Thus, the sample size of the study was calculated using the Raosoft sample size calculator (Raosoft Inc., Seattle, WA) with a 95% confidence interval and a margin of error of 5%. The study used a sample size of 425 participants.

Sampling technique

We adopted a simple random sampling technique to ensure every individual in the target population had an equal and independent chance of being selected for the study. We adopted this technique to eliminate bias and allow better generalization of research findings to the entire population from which the sample is drawn.

Data collection

The data collection tool (questionnaire) was developed using Google Forms (Google LLC, Mountainview, CA) and transmitted to single patient participants via social media platforms. The questionnaire was tested and exhibited satisfactory construct validity, strong reliability, acceptable internal consistency, and excellent content validity. An extensive review of the tools used and approved by other researchers and consultation with the experts to include only questions that address the problem under study. The survey was distributed via various social media platforms, including X (formerly known as Twitter), Meta (formerly known as Facebook), and WhatsApp. Each participant was asked to click on the consent statement before proceeding to complete the questionnaire. The questionnaire involved different sections, including a part with social demographic variables and sections with knowledge and awareness items.

Data analysis

The data collected were reviewed, coded, and then fed into IBM SPSS Statistics software for Windows, version 21 (released 2012; IBM Corp., Armonk, New York). Qualitative data were presented in frequencies and percentages, and quantitative data were presented in mean and standard deviation (SD). A suitable statistical test was used. A chi-square test was performed to test for differences in the proportions of categorical variables between two or more groups. P<0.05 was considered the cutoff value for statistical significance. All statistical tests used were two-tailed with an alpha level of 0.05. This was considered significant if the P-value was less than or equal to 0.05.

Ethical consideration

Ethical approval was obtained from the ethics committee at Taif University, Taif, Saudi Arabia (approval number: HAO-02-T-105). Information was provided to all participants prior to obtaining consent. Before collecting data, informed consent was obtained from the participants. Further, confidentiality was ensured for all interviewed participants. The data of each participant were taken in a re-identifiable manner. The data collected were stored electronically in electronic files, and no access was granted to anyone who was not a legitimate participant in the study.

## Results

A total of 425 participants from the Western province of Saudi Arabia were engaged in the assessment of their awareness and knowledge of bacterial septicemia (Table 1).

**Table 1:** Sociodemographic details (N=425) Social demographic characteristics are presented in frequencies (N) and proportions (%).

Variables	Category	N	%
Gender	Female	297	69.9
	Male	128	30.1
Nationality	Saudi	411	96.7
	Non-Saudi	14	3.3
Marital status	Unmarried	143	33.6
	Married	256	60.2
	Divorced	22	5.2
	Widow	4	.9
Place of residence	Taif	216	50.8
	Jeddah	124	29.2
	Makkah	42	9.9
	Yanbu	38	8.9
	Medina	5	1.2
Educational Level	No primary school education	2	.5
	Primary	7	1.6
	Middle	11	2.6
	Secondary	89	20.9
	Graduate	286	67.3
	Postgraduate	30	7.1
Average family income per month	<5,000 Saudi riyals	60	14.1
	5,000-10,000 Saudi riyals	126	29.6
	>10,000 Saudi riyals	239	56.2
Employment	Employed	199	46.8
	Not employed	127	29.9
	Student	99	23.3
Work in the healthcare sector	No	376	88.5
	Yes	49	11.5

The mean age of the participants was found to be 38.3±13.3 years. The sociodemographic characteristics showed that 297 (69.9%) of the participants were females, 411 (96.7%) were Saudi citizens, 256 (60.2%) were married, 216 (50.8%) were from Taif, and 124 (29.3%) were from Jeddah. Among the participants, 286 (67.3%) had graduate-level education, 239 (56.2%) had an average family income of >1,000 Saudi riyals per month, 199 (46.8%) were employed, and only 49 (11.5%) were from the healthcare sector.

Table 2 shows that 203 (47.8%) participants reported that they were aware of bacterial septicemia.

**Table 2:** Participants' awareness and knowledge of bacterial septicemia and their experience with it Experience related to bacterial septicemia is presented in proportions (%) and frequencies (N).

Variables	Category	N	%
Awareness of bacterial septicemia	No	222	52.2
	Yes	203	47.8
Source of awareness (n=203)	Internet	98	48.3
	Healthcare professionals	97	47.8
	Social media	78	38.4
	Friends and family	65	32.0
	TV/radio	32	15.8
	College/school	29	14.3
Risk factors of septicemia (n=203)	Lowered immunity	154	75.9
	Infection	80	39.4
	Severe illness	79	38.9
	Intubation and catheterization	53	26.1
	Poor personal hygiene	49	24.1
	Diabetes	40	19.7
	Burns and Injuries	39	19.2
Symptoms of septicemia (n=203)	Tachycardia (increased heartbeat)	58	28.6
	Difficulty breathing	42	20.7
	Lack of concentration	27	13.3
	Fever	64	31.5
Have you ever had bacterial septicemia?	No	422	99.3
	Yes	3	.7
Do you know someone with bacterial septicemia?	No	394	92.7
	Yes	31	7.3

The most common source of this awareness was the internet (98 participants, 48.3%), followed by healthcare professionals (97 participants, 47.8%), social media (78 participants, 38.4%), friends and family (65 participants, 32%), TV/radio (32 participants, 15.8%), and college/school curriculum (29 participants, 14.3%). In the assessment of risk factors for bacterial septicemia among those who were aware of it, the frequencies reported for each risk factor were as follows: burns and injuries: 39 participants (19.2%), diabetes: 40 participants (19.7%), intubation and catheterization: 53 participants (26.1%), infection: 80 participants (39.4%), severe illness: 79 participants (38.9%), and low immunity: 154 participants (75.9%). The awareness related to septic symptoms was as follows: increased heartbeat: 58 participants (28.6%), difficulty breathing: 42 participants (20.7%), lack of concentration: 27 participants (13.3%), and fever: 64 participants (31.5%). It was reported by only three (0.7%) participants that they had experienced bacterial septicemia, and about 31 (7.3%) knew someone with bacterial septicemia.

Table 3 shows the assessment of awareness based on different sociodemographic characteristics.

**Table 3:** Relationship between awareness and sociodemographic details Social demographic characteristics are presented in proportions (%) and frequencies (N). *A P-value <0.05 is considered statistically significant.

			Awareness of bacterial septicemia		Total	P-value*
			No	Yes		
Age	<=25 years	N	59	67	126	0.559
		%	46.8%	53.2%	100.0%	
	26-35 years	N	22	23	45	
		%	48.9%	51.1%	100.0%	
	36-45 years	N	52	45	97	
		%	53.6%	46.4%	100.0%	
	46-55 years	N	73	58	131	
		%	55.7%	44.3%	100.0%	
	>=56 years	N	15	10	25	
		%	60.0%	40.0%	100.0%	
Gender	Female	N	158	139	297	0.545
		%	53.2%	46.8%	100.0%	
	Male	N	64	64	128	
		%	50.0%	50.0%	100.0%	
Nationality	Saudi	N	215	196	411	0.865
		%	52.3%	47.7%	100.0%	
	Non-Saudi	N	7	7	14	
		%	50.0%	50.0%	100.0%	
Marital status:	Divorced	N	16	6	22	0.078
		%	72.7%	27.3%	100.0%	
	Married	N	137	119	256	
		%	53.5%	46.5%	100.0%	
	Unmarried	N	66	77	143	
		%	46.2%	53.8%	100.0%	
	Widow	N	3	1	4	
		%	75.0%	25.0%	100.0%	
Place of residence	Jeddah	N	58	66	124	<0.001
		%	46.8%	53.2%	100.0%	
	Makkah	N	23	19	42	
		%	54.8%	45.2%	100.0%	
	Medina	N	2	3	5	
		%	40.0%	60.0%	100.0%	
	Taif	N	131	85	216	
		%	60.6%	39.4%	100.0%	
	Yanbu	N	8	30	38	
		%	21.1%	78.9%	100.0%	
Educational level	Uneducated	N	2	0	2	0.461
		%	100.0%	0.0%	100.0%	
	Primary	N	5	2	7	
		%	71.4%	28.6%	100.0%	
	Secondary	N	42	47	89	
		%	47.2%	52.8%	100.0%	
	Middle	N	5	6	11	
		%	45.5%	54.5%	100.0%	
	Graduate	N	154	132	286	
		%	53.8%	46.2%	100.0%	
	Postgraduate	N	14	16	30	
		%	46.7%	53.3%	100.0%	
Average family income per month	<5,000 Saudi riyals	N	33	27	60	0.140
		%	55.0%	45.0%	100.0%	
	5,000-10,000 Saudi riyals	N	74	52	126	
		%	58.7%	41.3%	100.0%	
	>10,000 Saudi riyals	N	115	124	239	
		%	48.1%	51.9%	100.0%	
Job	Employed	N	105	94	199	0.045
		%	52.8%	47.2%	100.0%	
	Not employed	N	75	52	127	
		%	59.1%	40.9%	100.0%	
	Student	N	42	57	99	
		%	42.4%	57.6%	100.0%	
Work in the health sector	No	N	212	164	376	<0.001
		%	56.4%	43.6%	100.0%	
	Yes	N	10	39	49	
		%	20.4%	79.6%	100.0%	

It was observed that participants from Yanbu (30 participants, 78.9%) and Jeddah (66 participants, 53.2%) had significantly more awareness compared to other regions (P<0.001). Participants who were students (57 participants, 57.6%) had significantly higher awareness compared to others (P=0.045). Participants who worked in the healthcare sector showed significantly higher awareness (39 participants, 79.6%) compared to others (P<0.001).

Table 4 shows the assessment of the knowledge level in relation to participants’ sociodemographic characteristics.

**Table 4:** Relationship between knowledge regarding risk factors and symptoms and sociodemographic details Risk factors, symptoms, and social demographic characteristics are presented in proportions (%) and frequencies (N). *A P-value <0.05 is considered statistically significant.

			Knowledge level of risk factors and symptoms			Total	P-value*
			Good	Fair	Poor		
Age	<=25 years	N	3	3	61	67	0.535
		%	4.5%	4.5%	91.0%	100.0%	
	26-35 years	N	1	2	20	23	
		%	4.3%	8.7%	87.0%	100.0%	
	36-45 years	N	1	2	42	45	
		%	2.2%	4.4%	93.3%	100.0%	
	46-55 years	N	1	0	57	58	
		%	1.7%	0.0%	98.3%	100.0%	
	>=56 years	N	1	0	9	10	
		%	10.0%	0.0%	90.0%	100.0%	
Gender	Female	N	5	5	129	139	0.970
		%	3.6%	3.6%	92.8%	100.0%	
	Male	N	2	2	60	64	
		%	3.1%	3.1%	93.8%	100.0%	
Nationality	Saudi	N	7	6	183	196	0.251
		%	3.6%	3.1%	93.4%	100.0%	
	Non-Saudi	N	0	1	6	7	
		%	0.0%	14.3%	85.7%	100.0%	
Marital status	Divorced	N	3	3	71	77	0.705
		%	3.9%	3.9%	92.2%	100.0%	
	Married	N	3	4	112	119	
		%	2.5%	3.4%	94.1%	100.0%	
	Unmarried	N	1	0	5	6	
		%	16.7%	0.0%	83.3%	100.0%	
	Widow	N	0	0	1	1	
		%	0.0%	0.0%	100.0%	100.0%	
Place of residence	Jeddah	N	3	4	59	66	0.653
		%	4.5%	6.1%	89.4%	100.0%	
	Makkah	N	1	0	18	19	
		%	5.3%	0.0%	94.7%	100.0%	
	Medina	N	0	0	3	3	
		%	0.0%	0.0%	100.0%	100.0%	
	Taif	N	3	1	81	85	
		%	3.5%	1.2%	95.3%	100.0%	
	Yanbu	N	0	2	28	30	
		%	0.0%	6.7%	93.3%	100.0%	
Educational level	Primary	N	0	0	2	2	0.710
		%	0.0%	0.0%	100.0%	100.0%	
	Secondary	N	0	0	6	6	
		%	0.0%	0.0%	100.0%	100.0%	
	Middle	N	2	1	44	47	
		%	4.3%	2.1%	93.6%	100.0%	
	Graduate	N	5	4	123	132	
		%	3.8%	3.0%	93.2%	100.0%	
	Postgraduate	N	0	2	14	16	
		%	0.0%	12.5%	87.5%	100.0%	
Average family income per month	<5,000 Saudi riyals	N	1	1	25	27	0.970
		%	3.7%	3.7%	92.6%	100.0%	
	5,000-10,000 Saudi riyals	N	2	1	49	52	
		%	3.8%	1.9%	94.2%	100.0%	
	>10,000 Saudi riyals	N	4	5	115	124	
		%	3.2%	4.0%	92.7%	100.0%	
Job	Employed	N	3	3	88	94	0.743
		%	3.2%	3.2%	93.6%	100.0%	
	Not employed	N	1	1	50	52	
		%	1.9%	1.9%	96.2%	100.0%	
	Student	N	3	3	51	57	
		%	5.3%	5.3%	89.5%	100.0%	
Work in the health sector	No	N	5	4	155	164	0.212
		%	3.0%	2.4%	94.5%	100.0%	
	Yes	N	2	3	34	39	
		%	5.1%	7.7%	87.2%	100.0%	

The knowledge level related to risk factors and symptoms of bacterial septicemia was calculated using the total knowledge score obtained based on the correct responses for each of the risk factors and symptoms. Following this, knowledge level was calculated based on the percentages of knowledge scores, where a score >=75% was considered "good", 60%-74.9% as "fair", and <60% as "poor". The analysis showed that the majority (n=203, 93.1%) showed poor knowledge; good and fair knowledge was seen only in the minority (n=7, 3.4%). No statistically significant relationship was observed between the knowledge level and any of the sociodemographic characteristics (P>0.05).

## Discussion

According to the findings of the study, almost half of the participants (47.8%) reported being aware of bacterial septicemia. This indicates that a substantial portion of the sample population had some knowledge or understanding of the condition. Among those who were aware of bacterial septicemia, the most common source of awareness was the internet (48.3%), followed closely by healthcare professionals (47.8%). This suggests that individuals are actively seeking information about the condition through online resources and consulting with medical experts. Social media also played a significant role in raising awareness, as 38.4% of participants reported obtaining information from this source. When considering the risk factors associated with bacterial septicemia, the most frequently reported factors were low immunity (75.9%) and infection (39.4%). This highlights the importance of having a strong immune system and preventing and treating infections to reduce the risk of septicemia. Other reported risk factors include burns and injuries, diabetes, urinary catheters, and severe illness. In terms of symptoms, fever was the most commonly reported symptom associated with septicemia, with 31.5% of participants identifying it as a potential symptom. Increased heartbeat, difficulty breathing, and lack of concentration were also mentioned as symptoms, though to a lesser extent. It is worth noting that only 0.7% of participants reported personally experiencing bacterial septicemia, while 7.3% knew someone who had the condition. This suggests that while awareness of bacterial septicemia is relatively high, personal experience with the condition is relatively rare. These findings are similar to those of the study conducted by Van der Heide et al., who found in their study that limited personal encounters with bacterial septicemia and their reliance on the internet as their primary source of information may contribute to the lack of depth in both fields [12]. Jabaley et al. found that web analytics tracking should be employed to provide insight into information-seeking habits, given the prevalence of using the internet as a primary source of health information as a whole and sepsis information in particular [13].

The study findings also show that participants in urban and rural populations had significantly higher awareness compared to participants from other regions. Specifically, 78.9% of participants from Yanbu and 53.2% of participants from Jeddah had higher awareness levels. The significance level (P-value) is less than 0.001, meaning that the difference in awareness levels between regions is statistically significant. Similarly, participants who were students had significantly higher awareness compared to other participating groups. Specifically, 57.6% of students had higher awareness levels. The significance level (P-value) is 0.045, indicating that the difference in awareness levels between students and non-students is statistically significant. Furthermore, participants who worked in the healthcare sector showed significantly higher awareness compared to participants in other sectors. Specifically, 79.6% of participants in the healthcare sector had higher awareness levels. The significance level (P-value) is less than 0.001, suggesting that the difference in awareness levels between healthcare sector workers and non-healthcare sector workers is statistically significant. The findings are consistent with Rhee et al., who found in their study that increasing awareness about the risk factors of septicemia among healthcare workers led to improved infection control practices and a decrease in septicemia cases. Improved knowledge can decrease hospitalizations and medical costs associated with septicemia [14].

The results show that the majority of participants, 93.1%, had poor knowledge about the risk factors and symptoms of bacterial septicemia. Only a small percentage, 3.4%, had fair or better knowledge. This indicates that the majority of participants had low knowledge of the topic. This highlights the need for education and awareness campaigns to improve knowledge and understanding of septicemia and its risk factors and symptoms. The lack of significant relationships between knowledge level and sociodemographic characteristics suggests that knowledge about septicemia is lacking across different demographic groups. This further emphasizes the importance of targeted educational interventions to ensure that all individuals, regardless of their sociodemographic characteristics, have access to accurate information about septicemia. The significance level values (P>0.05) indicate that the lack of significant relationships between knowledge level and sociodemographic characteristics is not due to chance. The results suggest that sociodemographic characteristics such as age, gender, and education level do not have a significant impact on knowledge about bacterial septicemia. This highlights the need for targeted educational interventions to improve knowledge and awareness about bacterial septicemia, with a focus on reaching a wider audience. Further research could explore the potential reasons for the low knowledge levels observed in this study and develop effective strategies to improve knowledge and awareness about bacterial septicemia. Similarly, Haque et al., Johnson et al., and Gasim et al. noted that recognizing the symptoms of bacterial septicemia is essential for seeking prompt medical care. Septicemia can progress rapidly, leading to severe complications or even death if left untreated. Our findings indicated that participants had difficulty recognizing the early symptoms of septicemia. Symptoms like fever, rapid heart rate, confusion, and rapid breathing were frequently misinterpreted or overlooked. This lack of awareness could potentially delay timely medical intervention, leading to increased morbidity and mortality rates given the high incidence of sepsis in the region [15-17].

The study's sample was limited to one province in Saudi Arabia, potentially not representative of the entire country. It focused on quantitative data and did not explore qualitative aspects or cultural factors that could influence understanding. The research did not propose interventions to address the issue or investigate the impact of increased awareness on healthcare-seeking behavior or outcomes. However, the study provided a representative sample and baseline awareness levels for comparison. It also highlighted the need to raise awareness as a potential strategy for managing sepsis.

## Conclusions

In summary, this study reveals that while awareness of bacterial septicemia is relatively high among the sample population, knowledge about the risk factors and symptoms of the condition is low. The internet, healthcare professionals, and social media play significant roles in raising awareness about bacterial septicemia. Participants from Yanbu and Jeddah, students, and healthcare sector workers had higher awareness levels compared to other groups. However, overall knowledge levels about bacterial septicemia were poor across various demographic characteristics. This highlights the need for targeted educational interventions to improve knowledge and understanding of septicemia and its risk factors and symptoms among the general population. Further research is needed to identify the reasons for low knowledge levels and develop effective strategies to improve awareness about bacterial septicemia.
